# Anatomic Aspects of Inguinal Lymph Nodes Applied to Lymphadenectomy in Penile Cancer

**DOI:** 10.1155/2011/952532

**Published:** 2011-10-25

**Authors:** João Paulo Martins de Carvalho, Bruno F. Patrício, Jorge Medeiros, Francisco J. B. Sampaio, Luciano A. Favorito

**Affiliations:** Urogenital Research Unit, State University of Rio de Janeiro, 20551-030 Rio de Janeiro, RJ, Brazil

## Abstract

*Objectives*. To provide a better understanding of the distribution of inguinal nodes in order to prevent the complications of unnecessary and extended dissections in penile cancer. *Methods*. The bilateral inguinal regions of 19 male cadavers were dissected. Nodal distribution was noted and quantified based on anatomical location. The superficial nodes were subdivided into quarters as follows: superomedial, superolateral, inferomedial, and inferolateral. Statistical analysis was performed comparing node distribution between quarters using one-way analysis of variance (ANOVA), and the unpaired *T*-test was used between superficial and deep nodes. *Results*. Superficial nodes were found in all inguinal regions studied (mean = 13.60), and their distribution was more prominent in the superomedial quarter (mean = 3.94) and less in the inferolateral quarter (mean = 2.73). There was statistical significance between quarters when comparing the upper group with the lower one (*P* = 0.02). Nodes were widely distributed in the superficial region compared with deep lymph nodes (mean = 13.60 versus 1.71, *P* < 0.001). *Conclusions*. A great number of inguinal lymph nodes are distributed near the classical anatomical landmarks for inguinal lymphadenectomy, more prominent in upper quadrants.

## 1. Introduction

Penile cancer is an aggressive and mutilating disease that deeply affects the patient's self-esteem. Penile cancer is a rare neoplasia, particularly in developed countries. One of the world's highest prevalence rates is found in India, at 3.32 per 100,000 inhabitants, and the lowest incidence is among Jewish men born in Israel, with rates close to zero [1]. In the United States, the prevalence is 0.2 cases for each 100,000 inhabitants, whereas in Brazil, the national incidence of penile cancer 4.6 per 100,000 inhabitants (with a wide variation of 2.9 to 6.8 cases per 100,000 depending on the region), one of the world's highest rates of this neoplasia [2, 3]. 

The most common sites of penile cancer metastasis are the superficial and deeper nodes of the inguinal and iliac region. Patients have inguinal groin masses in 58% of cases, and 40% have positive metastasis, even in small cancers such as T1C and T2 [4]. 

Extended Inguinal lymphadenectomy is the most useful and commonly performed surgery for staging and to cure inguinal metastasis in penile cancer cases. Although it is a widespread technique, postoperatory complications often occur, such as cutaneous flap necrosis, lymphedema, and vascular lesions, including in the saphena magna ligature, with subsequent deeper venous thrombosis of the affected limb [5].

Knowledge of the inguinal region and node distribution is important to prevent such complications and to diminish the morbidity caused [6, 7]. The venous drainage of the inguinal region occurs mainly through the external pudendal, superficial circumflex iliac and saphena magna veins. The inguinal nodes are one of the major lymphatic blocs of the human body. They are responsible for drainage of the inferior limbs, genitalia, posterior perineum, and inferior extremity of the abdominal wall. The nodes can be subdivided into two groups: superficial, placed just below the subcutaneous, and deeper inguinal nodes, close to the femoral vein under the fascia lata. The latter are drained mainly by the external iliac nodes [8, 9].

The best technique for inguinal dissection in penile cancer should include all the nodes situated in the inguinal region. Studies focused on anatomical landmarks for penile cancer are rare. The aim of this study is to provide a better understanding of node distribution, by describing and quantifying the superficial and deeper inguinal nodes, to help prevent the complications of unnecessary and extended dissections.

## 2. Material and Methods

The present work was approved by the bioethics committee of our institution and was in accordance with the ethical standards of the committee on human experimentation. From May 2010 to March 2011, we analyzed 19 fresh cadavers (38 inguinal regions) from males aged 23 to 53 years old (mean = 32) who had been submitted to dissection of the inguinal region. All cadavers were obtained through donations from the university, and none of the decedents were trauma victims.

**Figure 1:**
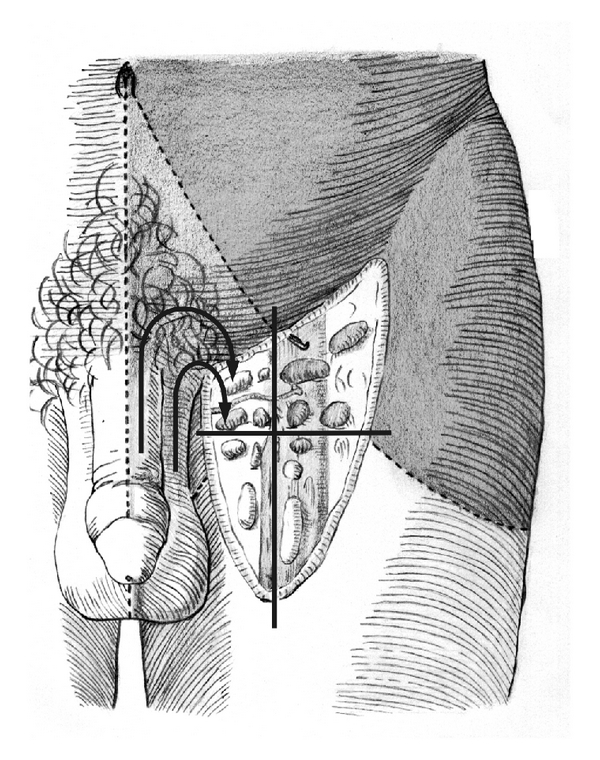
Schematic drawing of lymphatic drainage of inguinal nodes. There is a cross with its middle point in the saphena hiatus. The penile and scrotum lymphatic drainage is performed, by the upper internal quarter (arrows).

The superficial inguinal region was divided into four quadrants, by drawing two perpendicular lines over the saphenofemoral junction (Figure 1): superomedial, superolateral, inferomedial, and inferolateral. The inguinal nodes were divided into superficial group and deeper group (situated medially to the femoral vein and below the fascia lata). All the dissections were performed by the same surgeon, and the nodes were quantified as soon as they were noted, by two different observers.

Statistical analysis was performed based on the superficial node distribution in quarters: superomedial, superolateral, inferomedial, and inferolateral, using one-way analysis of variance (ANOVA) and the unpaired *T*-test. Statistical significance was considered with a *P* value under 0.05.

## 3. Results

**Figure 2:**
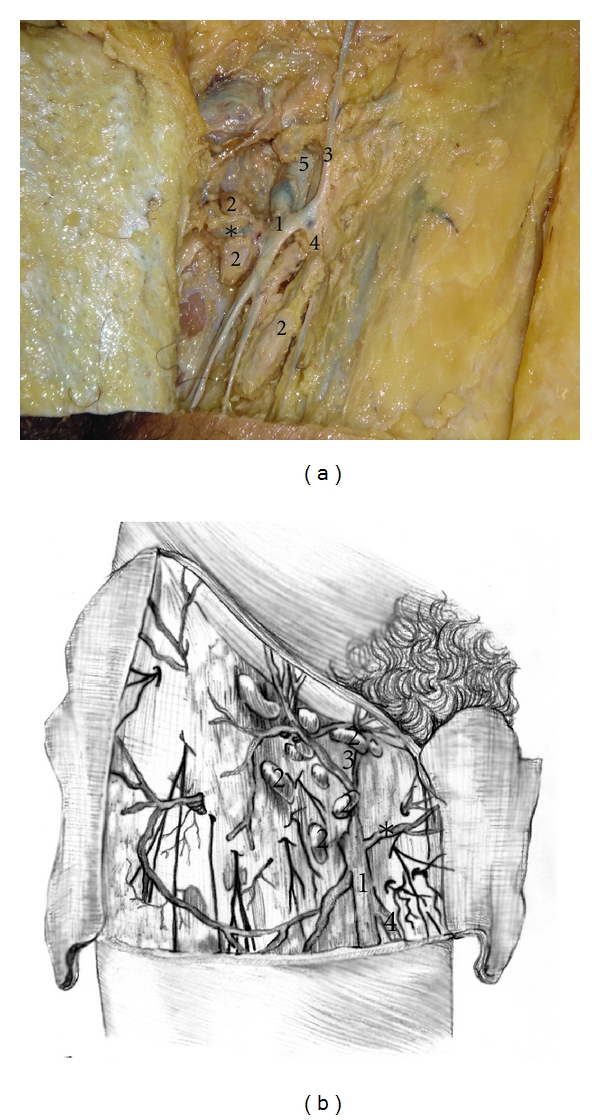
(a) Inguino crural dissection in a formalin-preserved cadaver. The subcutaneous tissue has been removed and the following structures can be identified: (1) saphena magna vein; (2) superficial lymph nodes; (3) superficial epigastric vein; (4) accessory saphena vein; (5) *-external pudendal vein. (b) Schematic draw of the superficial inguinal region and nodes.

The dissections preserved the saphenofemoral junction and all the tributaries of the saphena magna vein, which permitted adequate visualization of the nodes and their classical anatomical landmarks (Figure 2).

Superficial nodes were found in all inguinal regions, with a variation of 5 to 17 nodes (mean = 13.60) and the distribution was more prominent in the superomedial quarter (mean = 3.94) and less in the inferolateral quarter (mean = 2.73). There was a statistical significance between quarters when comparing the upper groups with the lower ones (*P* = 0.02). There was no statistical difference between the superomedial (mean = 3.94) and superolateral (mean = 3.76) and between the inferomedial (mean = 3.15) and inferolateral quarter (mean = 2.73).

**Figure 3:**
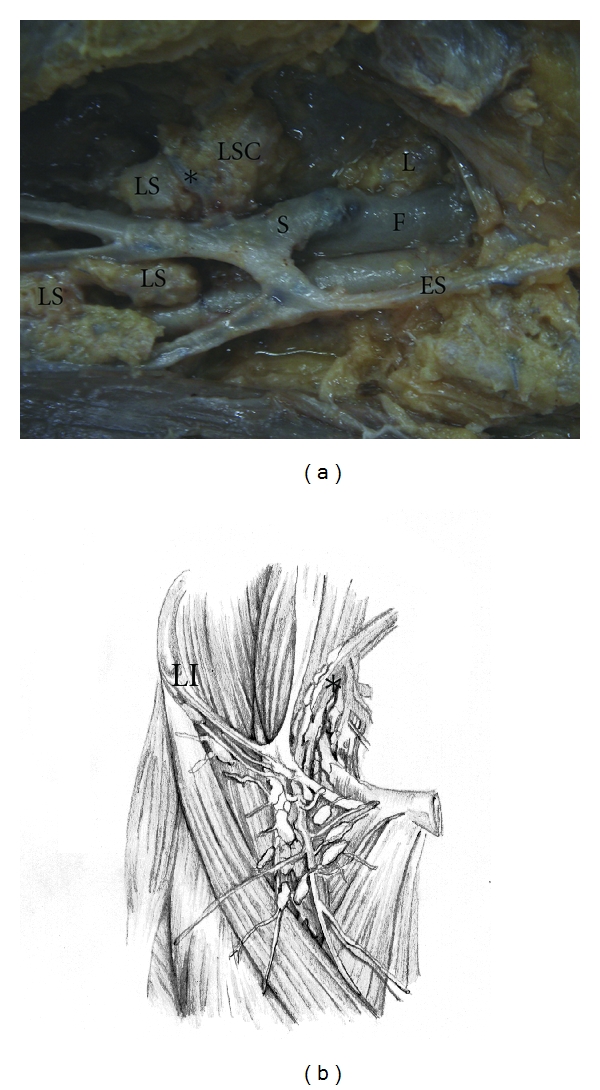
(a) Inguino crural dissection in formalin-preserved cadaver. The superficial nodes (SN) remain in their original position. In this specimen, just one deep inguinal lymph node is located medially to the femoral vein (F). S: saphena magna vein; SE: superficial epigastric vein; *-external pudendal vein; SSN: superficial sentinel node, as previously described by Cabanas [10, 11]. (b) Schematic draw with superficial and deep nodes of the inguinal region and the iliac nodes.

**Figure 4:**
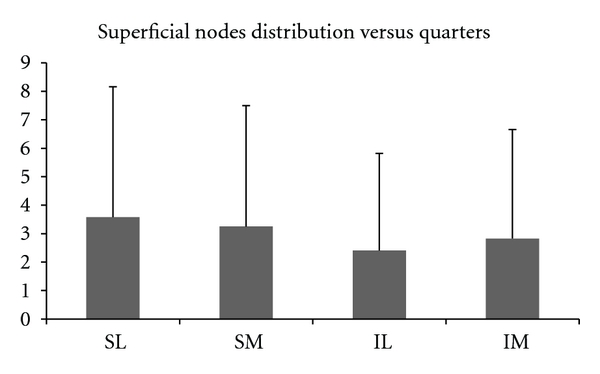
Average superficial nodes distribution versus quarters; SL: superolateral; SM: superomedial; IL: inferolateral; IM: inferomedial quarters. No statistically difference between samples.

Deeper nodes were not found in all of the cadavers, and their number ranged from 0 to 5 (mean = 1.71) (Figure 3). Nodes were widely distributed in the superficial region compared with deep lymph nodes (mean = 13.60 versus 1.71, *P* < 0.001). The Figure 4 shows the average superficial nodes distribution versus quarters in the 19 cadavers.

## 4. Comments

Penile lymphatic drainage parallels venous drainage, with a superficial system that drains the skin and a deeper system that drains the glans and corporal bodies. The superficial inguinal nodes are located just below the inguinal ligament and extend trough 4-5 cm of the saphenous hiatus. They are distributed in quarters set from the anastomosis between the saphena magna and femoral veins [9]. The deeper inguinal nodes are located just below the fascia lata and medially to the saphena vein. Although small in number, these nodes are of extreme importance, since their venous drainage occurs through the superficial iliac veins [8, 9]. 

Cabanas [11] described the lymphangiography patterns in a large number of patients with penile cancer. Injection into the lymphatics of the dorsal penile vessels consistently drained into a node located anterior or medial to the superficial epigastric vein and superomedial to the epigastric saphenous junction, with subsequent drainage into the deep inguinal and iliac chains. All patients in this study with metastatic disease demonstrated involvement of this sentinel node [9–11].

To quantify the nodes in the present study, we performed it in quarters [12] rather than as previously described by Ruviere [13], Daseler et al. [14], and Leijte et al. [15] who describe a fifth inguinal region overlying the saphenous femoral junction. The central region under anatomical landmarks is difficult to delimit, so nodes dissected in this area could belong to another anatomical region. In our point of view, the use of quadrants facilitates dissection and quantification of the nodes in the inguinal region.

The first technique described for inguinal lymph-node dissection (ILND) for penile cancer found in Medline resources was attributed to Zenker and Pichlmaier in 1966 [16]. Since then, hundreds of modifications and new approaches have been published. Fegen and Persky [17] first associated the success of a complete inguinal dissection with an increase in penile cancer survival rates.

In the 1970s, new methods of staging were developed and the use of lymphangiograms and fluorescein increased its applicability, especially in the field of penile cancer [10, 18]. The reason for seeking new methods was to avoid overly extensive inguinal node dissections and related procedures. Complications of these techniques cause high rates of morbidity and death [19, 20], justifying the use of sentinel lymph-node dissection [21].

In a recent publication by Zhu et al. [22], the incidence of positive nodes obtained from inguinal dissection in patients with penile cancer was highest in the medial block of nodes. Although the authors used different anatomic landmarks, the results are similar to the ones presented in this study.

The challenge of prophylactic ILND is to perform free margin cancer surgery with complete resection of all positive LNs without promoting an increase in morbidity. The team should attempt to perform the lymphadenectomy in regions with highest node distribution, without leaving any suspect mass [23].

Superficial node distribution was observed to be more prevalent in the upper quarters of the inguinal region, but it is important to note that the anatomical basis for this study is not correlated to any previous published study. Our group counted from 5 to 17 lymph nodes (mean = 13.60). Ruviere described 4 to 25 nodes in the superficial group (mean = 8.25), but did not describe the anatomical regions. 

After performing the superficial inguinal node dissection, the surgeon should be more cautious in the upper quarters, using the superficial femoral vein as an anatomical landmark, since the chance of encountering lymph nodes is greater. 

In contrast, deeper inguinal nodes, not encountered in all cases, were more prevalent in the medial region of the femoral vein, using the deep saphena vein and fascia lata as anatomical landmarks. These results are important, since despite the challenging distribution and location, this node group should be completely resected when performing inguinal nodes dissection for penile cancer in order to perform the staging of distant nodes in the same surgery and not to leave any suspect lymph nodes [5].

## 5. Conclusions

Our group confirmed that a great number of inguinal lymph nodes are distributed near the classical anatomical landmarks for ILND, as superficial and deeper femoral veins, inguinal ligaments, and fascia lata. The nodes were more prominent in the upper quadrants of the superficial inguinal lymph nodes. When performing prophylactic ILND using these criteria, less extensive surgery should be performed, with adequate node resection, including the superficial and deeper groups.
